# Terpyridine-Containing π-Conjugated Polymers for Light-Emitting and Photovoltaic Materials

**DOI:** 10.3389/fchem.2020.592055

**Published:** 2020-10-14

**Authors:** Pan Liu, Ganhui Shi, Xuegang Chen

**Affiliations:** Key Laboratory of Rubber-Plastic of Ministry of Education (QUST), School of Polymer Science and Engineering, Qingdao University of Science and Technology, Qingdao, China

**Keywords:** terpyridine, light-emitting materials, photovoltaic, metallo-polymers, energy conversion

## Abstract

2,2′:6′,2″-Terpyridine (tpy) is a versatile moiety used in the construction of small novel molecules or polymers. Extending or coupling tpy with π-conjugation structures can result in interesting optoelectronic properties. This mini-review summarizes the significant progress made over the past decades in the study of tpy-containing π-conjugated polymers and their application in light-emitting and photovoltaic materials. When coordinated with metal ions, tpy exhibits immense potential for the synthesis of metallo-supramolecular or metallo-polymer materials. Therefore, tpy-based metallo-polymers are the primary focus of this study. Selected examples will be reviewed with a special emphasis on the properties of these functional systems, which can consequently help further their application in light-to-electricity or electricity-to-light conversion fields.

## Introduction

2,2′:6′,2″-Terpyridine (tpy) ligands are effective coordinating agents and key building blocks in supramolecular chemistry and materials because the 4′-position of the central pyridine ring can be easily substituted. Since tpy can yield stable complexes with d-block metal ions, it is widely used in linear polynuclear metal complexes, grids, and metallomacrocycles, as well as metal coordination polymers (Hofmeier and Schubert, 2004; Puntoriero et al., 2008; Wild et al., 2011; Schultz et al., 2012; Chakraborty and Newkome, 2018; Schmolke et al., 2019; Qian et al., 2020). Nowadays, energy conversion materials play a vital role in modern life and industry. Among these, photoelectric materials, which include both electricity-to-light and light-to-electricity conversion materials, are especially important (Deng et al., 2018, 2019; Zhang et al., 2019, 2020). The advantages of tpy-based metallo-polymers, increased coordination with transition metal ions, easily modifiable molecular structure, performance, excellent electrochemical properties, and good thermal stability, render them appropriate candidates for application in photoelectric materials and devices. Here, we review the most important results that deal with the synthesis of π-conjugated polymers containing tpy moieties, with an emphasis on metallo-polymers, and their application in light-emitting and photovoltaic materials and devices.

## Light-Emitting Materials

### Terpyridine in the Side Chain

As a tridentate ligand, tpy can form stable complexes with many metal ions, including transition metal ions and rare earth ions, and is therefore used in light-emitting materials. Several organic light-emitting diodes (OLEDs) based on tpy complexes were fabricated and evaluated, and the tpy units introduced into the side chain of the functional polymers were usually coordinated with metals. In 2011, Dumur et al. (2011) published a study about the synthesis of two random copolymers bearing pendant mixed-ligand orthometallated tpy-based cationic Ir(III) complexes and their application emitters in light-emitting electrochemical cells (LECs) or as dopants in OLEDs. The polymers were obtained from the copolymerization of tpy-containing monomers and styrene through nitroxide-mediated polymerization. The resulting polymers possessed good film-forming properties, which were attributed to the polystyrene structures in the main chain. However, the absence of charge carriers and phase segregation made it difficult to inject holes, leading to the development of pool emission properties, such as brightness (70 cd·m^−2^) and efficiency (5 cd·m^−2^). Furthermore, the results showed that in cationic metallo-polymers, a minimum iridium concentration of 5% is necessary for light emission.

Lanthanides, or rare earths, compose the 5d block of the periodic table. Most Ln(III) ions are luminescent and play a vital role in lighting and light conversion (Ozawa and Itoh, 2003; Kotova et al., 2018), and tpy and tpy-like ligands showed several interesting luminescent properties when combined with lanthanide ions (Beck and Rowan, 2003). Ghosh et al. (2015) reported the multicolor luminescent properties of elastin-like polymers (ELPs) with tpy derivatives incorporated into their side chains. The tpy ligand was conjugated with an ELP through the amidation reaction between 4-amino-functionalized-tpyanda carboxyl group in the ELP. The tpy moieties acted not only as ligands for complexation with lanthanide ions [including Eu(III), Tb(III), Dy(III), Er(III), and Nd(III)] but also as an antenna. Consequently, they showed strong light absorption and then transferred the energy to the emitting metal ions, which resulted in high emission efficiency and sharp emission peaks. The photoluminescence (PL) spectra ranged from the visible to the near-infrared (NIR) regions (1,450–1,600 nm), and the direct excitation wavelength (λ_exc_) of the ligands was 395 nm. Since the lanthanide ions have no absorption at 395 nm, the NIR luminescence was associated with the intramolecular energy transfer from the photosensitizing organic ligands. Ru et al. (2014) also reported a similar energy transfer from polymer ligands to the emitting level of the rare earth ions in luminescent materials of Eu(III) coordinated by a tpy-functionalized poly(ionic liquid). The emission spectrum excited at 340 nm showed the characteristics of Eu^3+^ emissions in the 570–725-nm range without any broad emission band from the polymers. Yang et al. (2013) reported three nearly monochromatic red electroluminescent (EL) chelating polymers containing carbazole segments and tpy moieties, which serve as neutral ligands to coordinate with the Eu(2-thenoyltrifluoroacetonate)^3^ complex. Electroluminescence studies demonstrated that the EL devices of Eu-polymers based on tpy as a neutral ligand exhibited red bright emissions. Moreover, a maximum luminance of 68.2 cd·m^−2^ was recorded for the double-layer devices.

In addition to their advantageous coordination with metal ions, π-conjugated tpy units in the side chains can also bring about various interesting structures and their corresponding properties. In 2017, Wang et al. (2017) reported about the preparation of a novel white-light-emitting fluorescent polymeric material via aggregation of a single fluorescent chromophore through intermolecular quadruple hydrogen bonding. The material was later used as a gel to fabricate a protected quick response code. Interestingly, the pyridinium salt monomer, which was a donor–acceptor structure and attached to the tpy unit, emitted blue light, whereas the supramolecular polymer gel created by radical polymerization showed strong white fluorescence under UV light. Two fluorescence emission peaks were observed at ~474 and 571 nm, which was the result of the aggregation of the chromophore and the formation of a charge transfer complex based on the tpy moieties in the polymers' side chain.

### Terpyridine as Part of the Main Chain

**Figure 1:**
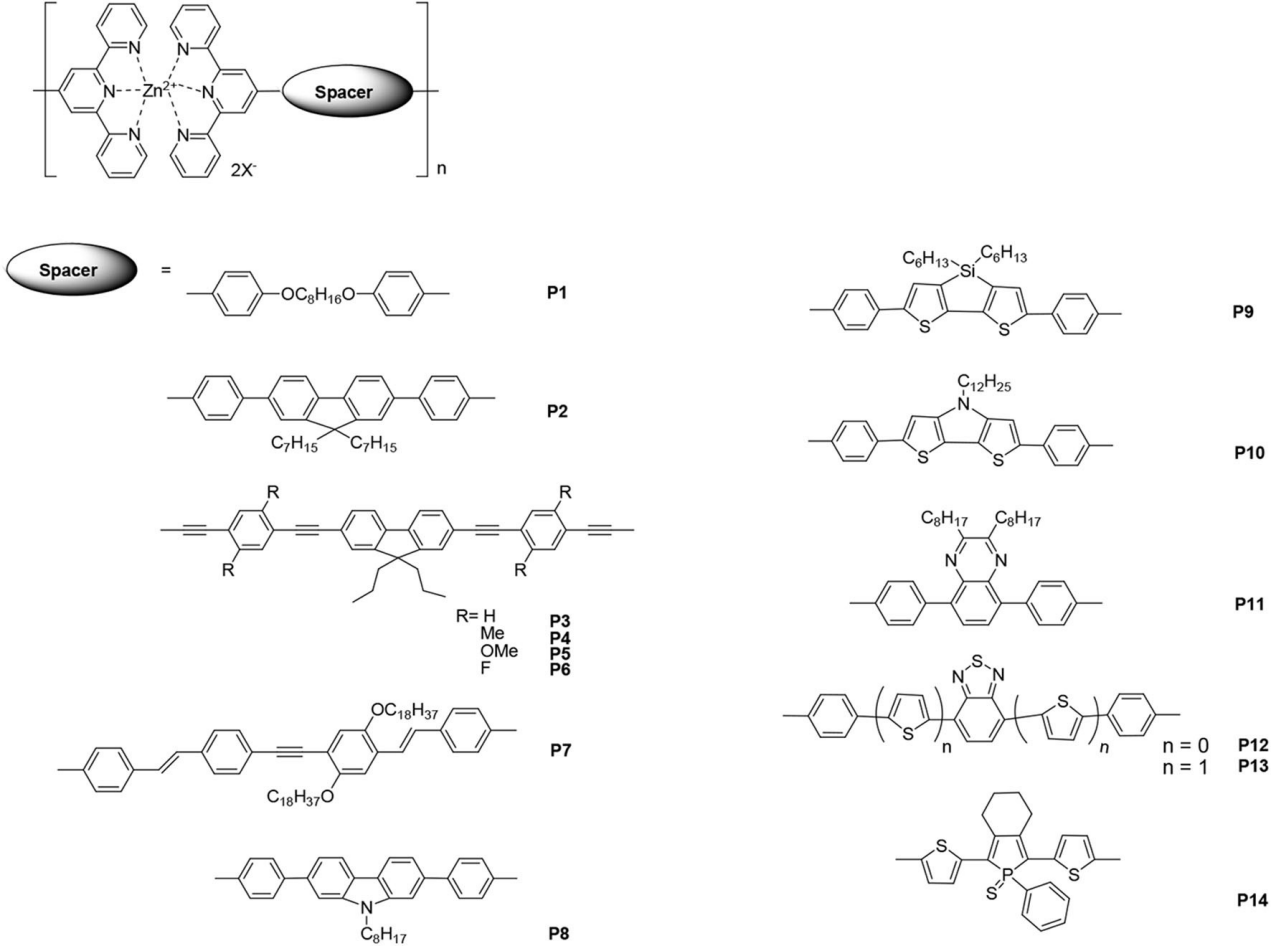
The selected bis(terpyridines) with different spacers as the building blocks in linear Zn(II) polymers.

The linear polymers containing tpy units are discussed in this section. The metallo-polymers produced by the introduction of metal ions into the π-conjugated main chains could possess interesting optical properties. Zinc ions are the most common metal ions used for light-emitting materials because complexation with zinc ions will not cause fluorescence quenching and may even enhance fluorescence. Figure 1 lists the selected zinc–tpy-based polymers that are employed in light-emitting materials.

Emission properties such as wavelength and/or efficiency can be regulated by modifying the conjugated spaces connected with bis(tpys). In 2003, Yu et al. (2003) reported the synthesis of a family of self-assembled zinc–tpy-based polymers, which emit violet to yellow light, via self-assembly reactions. The spaces between the bispyridines were involved in flexible and rigid structures. Most of the resulting metallo-polymers exhibited higher PL quantum yields compared with that of monomers, and the emission was attributed to intra-ligand ^1^(^*^π-π) fluorescence. Among these polymers, polymers **P1** and **P2** were fabricated into an EL device with a single layer configuration. Blue (CIE: *x* = 0.15, *y* = 0.21) and yellow (CIE: *x* = 0.46, *y* = 0.50) emissions with a maximum luminance of 1,700 and 2,380 cd·m^−2^ were obtained, respectively. The results indicated that these Zn(II)-containing metallo-polymers are promising light-emitting materials for polymer light-emitting devices (PLEDs). Chen and Lin (2007) reported similar metallo-polymers **P3**–**P6**, in which central π-aromatic 9,9-dipropylfluorenes were linked to tpy units through phenylene/ethynylene fragments. Overall, the different substituents on the metallo-polymers caused adjustable photophysical and thermal properties. The decomposition temperatures (*T*_d_) of the monomers under a nitrogen atmosphere ranged from 297 to 351°C, while those of the corresponding polymers ranged from 325 to 410°C. The EL emission colors of polymers **P4**, **P5**, and **P6** were yellow to orange (at a bias voltage of ~10 V), and the turn-on voltages of all devices were 6 V. The polymer **P5** showed the best power efficiency, external quantum yield, and brightness, which were 0.33 cd·A^−1^ (at 14 V), 1.02%, and 931 cd·m^−2^ (at 14 V), respectively. In another study, a series of main-chain metallo-polymers, e.g., **P7**, was prepared through the self-assembly of rigid-linear π-conjugated bis(tpy) monomers with Zn(II) ions (Winter et al., 2009). Solution-processing methods such as spin coating and inkjet printing were applied to prepare thin homogeneous films for photophysical studies; the metallo-polymer **P7** exhibited intense yellow PL emission [PL quantum yield of 0.82 in CHCl_3_/(CH_3_)_2_NC(O)H]. Compared with the corresponding PL spectra of **P7** in the solid state, a red shift was observed at the emission maximum (~30 nm). Furthermore, the EL performances displayed the potential of these polymers as light-emitting materials for PLEDs.

Li et al. (2016) synthesized three new building blocks containing the tpy electron-acceptor motif and the electron-donor fused-ring carbazole, dithienosilole, and dithienopyrrole motifs. The introduction of Zn(II) initiated the self-assembly polymerization that led to the formation of their corresponding metallo-polymers. The PL maxima of polymers **P8**, **P9**, and **P10** in the film were 443, 553, and 586 nm, respectively. Compared with the monomeric building blocks, the sharp red shifts in the polymers' PL arose from the incorporation of the transition metal ion into the backbones of the polymers, which also enhances the electron-deficient ability of the tpy moieties. Consistent with their photophysical properties, the modification by spacers with strong electron-donating ability increased the highest occupied molecular orbital (HOMO) level, whereas coordination with Zn(II) led to lower unoccupied molecular orbital (LUMO), which resulted in light-emitting materials with narrow band gaps (i.e., 2.07, 1.97, and 1.56 eV for **P8**, **P9**, and **P10**, respectively).

There have also been studies about introducing certain dye segments into the main chains of tpy-based metallo-polymers. Wild et al. (2013) described a variety of Zn(II) bis(tpy) metallo-polymers with a spacer dye moiety surrounded by thiophene donors. The tuning of the photophysical properties of these polymers can be obtained by systematically modifying the dye and the conjugation length. Owing to the dynamic nature of the Zn(II) complex, a great number of emission colors can be obtained depending on the energy transfer processes used and by carefully regulating the mixing ratio of blue **P11** (λ_PL_ = 443 nm), green **P12** (λ_PL_ = 503 nm), and red **P13** (λ_PL_ = 606 nm) light-emitting metallo-homopolymers. Moreover, in order to screen the thin-film photophysical properties in a reproducible and material-saving manner, the inkjet printing technique was employed to separately print every single color and subsequently print one solvent layer to assemble statistical copolymers.

Contrarily, Vitvarová et al. (2017) synthesized a novel building block that comprised a substituted phosphole ring surrounded by two thiophene rings with tpy fragments as end-groups. It was then coordinated with metal ions such as Co^2+^, Cu^2+^, Fe^2+^, Ni^2+^, and Zn^2+^ to produce metallo-supramolecular polymers (MSPs). The MSP with Fe^2+^ ion couplers showed very slow constitutional dynamics and a strong band of metal-to-ligand charge transfer (MLCT) transitions, whereas the MSPs with Co^2+^, Cu^2+^, and Ni^2+^ ion couplers exhibited variable dynamics and no MLCT bands. All of these MSPs showed luminescence quenching. However, the zinc-containing polymer **P14**, which exhibited very fast constitutional dynamics, showed the highest luminescence intensity at ~641 nm of PL maximum (luminescence quantum efficiency Φ = 0.5) without any MLCT transitions. Evidently, luminescence or quench is not related to MLCT transitions.

Other metals such as cadmium (Chen et al., 2007) and rare earth metals (Sato and Higuchi, 2012) can also be employed in the construction of tpy-based luminescent MSPs. Furthermore, several novel structures such as copolymers containing electron-withdrawing and electron-donating building blocks (Schlütter et al., 2010) and 2D (Fermi et al., 2014; Yin et al., 2018) polymers have been reported in the past decade.

## Photovoltaic Materials

**Figure 2:**
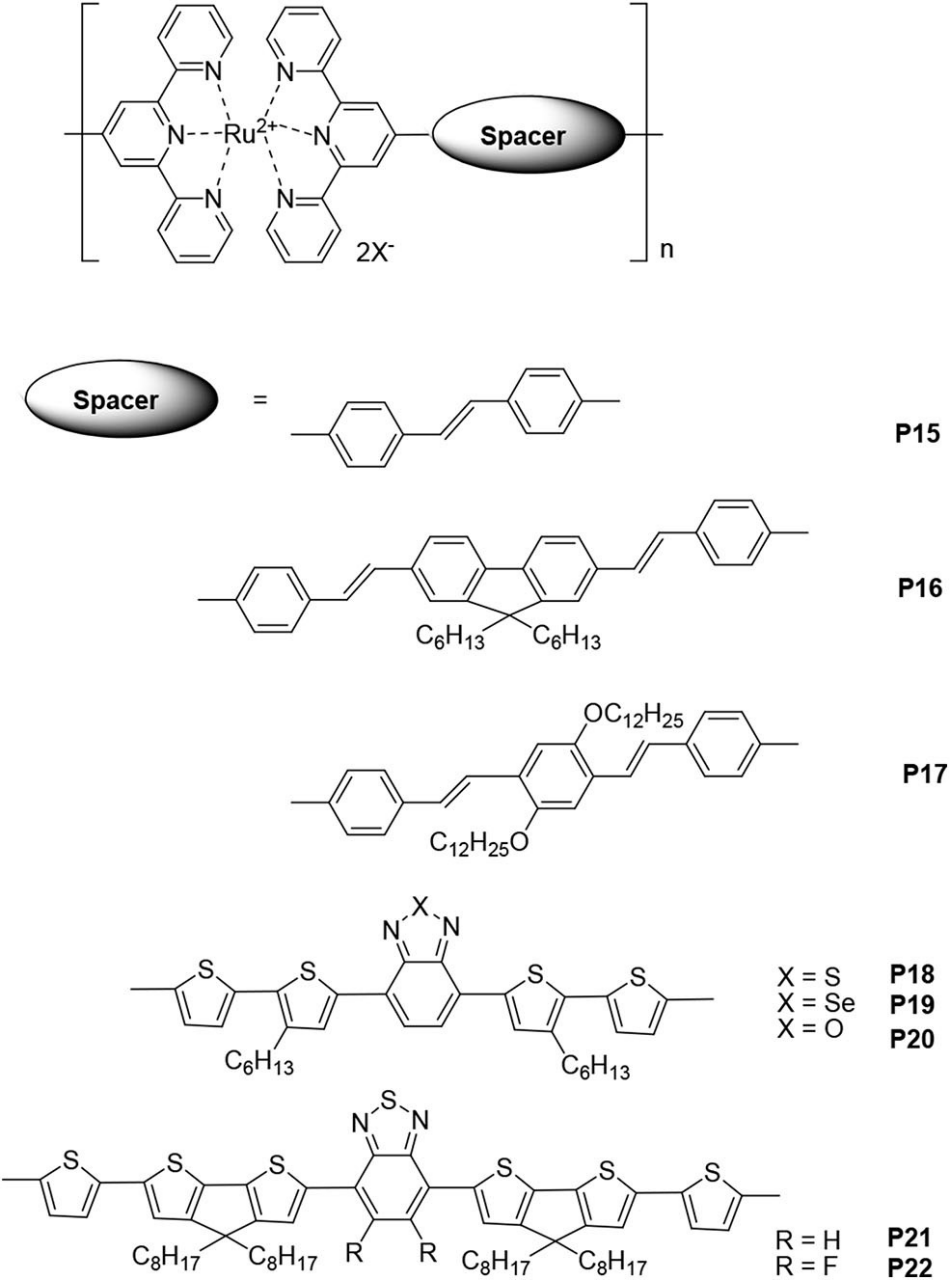
The selected ruthenium-supramolecular polymers containing conjugated bridges or donor–acceptor structures and terpyridines that are used in photovoltaic cells.

Owing to their flexibility, low cost, and ease of fabrication and manipulation, organic photovoltaic cells (OPVs) have garnered much attention in recent years (Hains et al., 2010). Studies on OPVs aim to improve the range of absorption, efficiency, charge transport, and stability of organic materials and cell devices. One of the key ways to achieve this is by improving the spectral match between solar light and organic sensitizing materials. Since ruthenium complexes exhibit reversible Ru(II)/Ru(III) redox processes and MLCT transitions in the range 500–600 nm, organic materials based on ruthenium polypyridine complexes are widely used in OPVs (Zakeeruddin et al., 2002; Numata et al., 2013). Cheng et al. (2008) firstly reported synthesis and photovoltaic performances of the metallo-polymers based on tpy units. Here, selected polymers containing Ru(II)-tpy chromophores, which are well-known photoactive moieties, will be discussed. In these metallo-polymers, the occurrence of tpys in side chains is rare, so most of them have Ru(II)-tpy as a part of their main chains (Figure 2).

Vellis et al. (2008) reported a new series of conjugated building blocks that bear terminal tpy moieties, which were connected to the central cores that were substituted with fluorine or phenylene segments through a vinylene bond. Coordinated with Ru(II), the metallo-polymers **P15**, **P16**, and **P17** can be obtained without any catalyst. In addition, a star-shaped metallo-polymer based on triphenylamine was also synthesized. The absorption range of the Ru(II)-tpy-based metallo-polymers was 300–550 nm, which was a result of the ligand-centered (LC) transitions at shorter wavelengths and MLCT transitions at longer wavelengths. In order to evaluate the photovoltaic properties of these metallo-polymers, bulk heterojunction devices with the structure ITO/PEDOT/Ru(II)-polymers/P3HT:PCBM (1:1 w/w)/Ca/Al were also fabricated. Among these polymers, polymer **P15** exhibited the highest power conversion efficiency (PCE) value (0.71%). The short-circuit current (J_sc_), open-circuit voltage (V_oc_), and fill factor (FF) values were 4.2 mA·cm^−2^, 0.48 V, and 35%, respectively. It needs to be noted that the preliminary results were obtained without optimization of device fabrication conditions.

Introducing electron-donor and electron-acceptor structures can decrease the HOMO level and increase the absorption range and thereby increase the photovoltaic cell performance. Padhy et al. (2011) reported about Ru(II)-supramolecular polymers **P18**, **P19**, and **P20**. The ideal HOMO/LUMO levels, reduced energy gaps, and broad absorption range (300–750 nm) were obtained by introducing donor–acceptor structures, such as electron-acceptor benzothiadiazole, benzoselenodiazole, and benzoxadiazole units and electron-donor thiophene units, into the main chains of the metallo-polymers. The optimal photovoltaic cell device, based on the blended polymer **P18**:PCBM = 1:1 (w/w), had PCE = 0.45%, V_oc_ = 0.61 V, J_sc_ = 2.18 mA·cm^−2^, and FF = 34.1%. Feng et al. (2013) synthesized bis(tpy) ligands with cyclopentadithiophene-benzothiazole conjugated bridges, and supramolecular polymers (**P21** and **P22**) containing Ru(II) were obtained via a supramolecular self-assembly process. In the main chains of these polymers, the cyclopentadithiophene and thiophene units were used as electron donors (D), and benzothiazole and fluorinated benzodiazole units were used as electron acceptors (A). The low-lying HOMO levels for **P21** and **P22** were −5.22 and −5.27 eV, respectively, and their electrochemical band gaps were 1.60 and 1.58 eV, respectively. Owing to the stronger π-π stacking, a result of the F-H, F-S, and/or F-F interactions, polymer **P22** exhibited a mobility one order of magnitude higher than that of polymer **P21**. The hole mobilities of **P21** and **P22** were 7.5 × 10^−6^ cm^2^·V^−1^s^−1^ and 2.8 × 10^−5^ cm^2^·V^−1^s^−1^, respectively. The photovoltaic device fabricated with polymer **P22** and with ITO/PEDOT:PSS/polymer:PC_71_BM/Ca/Al structure exhibited the highest PCE (2.66%) with a V_oc_ = 0.73 V, J_sc_ = 7.12 mA·cm^−2^, and FF = 0.51. The improved performance was a result of the introduction of the fluorine atom that then caused the low-lying HOMO level, narrow band gap, high hole mobility, and fine phase separation. Thus, the results of photovoltaic devices based on metallo-polymers are definitely inspiring.

Furthermore, a number of metallo-copolymers, block copolymers, and random copolymers have been reported in a few studies (Duprez et al., 2005; Padhy et al., 2012). Although their photovoltaic performances are not excellent, the incorporated π-conjugated units have several interesting properties. In fact, metal ions were not necessary to construct novel conjugated main chains. A recent study described the synthesis of various conjugated polymers containing only tpy segments, in which tpy-substituted carbazole (TPCz) was used as the electron donor, followed by the fabrication and evaluation of photovoltaic cells (Wang et al., 2020).

## Perspective

The introduction of metal ions to the main chains or side chains of polymers imparts them with a variety of properties. In light-emitting devices, different combinations of metals and ligands emit different colors, and they have high EL efficiency and brightness. Conversely, compared with the traditional polymers, in which the structures are connected by covalent bonds, the preparation of metallo-polymers has mild reaction conditions and does not require any catalyst, and the polymer structures are definite and easy to modify. Consequently, the HOMO/LUMO levels, energy gaps, spectral match with solar light, morphologies of polymer films, and photovoltaic device performances can be improved by carefully designing bridges connected to bis(tpy). Therefore, it is possible to predict that an increasing number of tpy-containing complexes or polymers can be synthesized in the future, that the unknown and puzzling problems involved in supramolecular chemistry of tpy and metal ions can be elucidated, and that better performances for light-to-electricity or electricity-to-light conversion materials and devices can be achieved.

## Author Contributions

PL and GS collected, arranged documents, and wrote some paragraphs for draft. XC prepared full text and revised. All authors contributed to the article and approved the submitted version.

## Conflict of Interest

The authors declare that the research was conducted in the absence of any commercial or financial relationships that could be construed as a potential conflict of interest.

## Footnotes

^1^Singlet excited state.
